# Guide of the Practical Physician, or General Summary of Internal Pathology and Therapeutics, Clinically Applied

**Published:** 1845-10

**Authors:** 


### ART. XIV.

1. *Guide du Médecin Praticien, ou Résumé général de Pathologie Interne et de Thérapeutique appliquées.* Par F. L. J. VALLEIX, Médecin du Bureau central des Hôpitaux de Paris, etc. Tome I.—*Paris,* 1842.

*Guide of the Practical Physician, or General Summary of Internal Pathology and Therapeutics, clinically applied.* By F. L. J. VALLEIX, Physician to the Central Bureau of Hospitals of Paris. Vol. I.—*Paris,* 1842. 8vo, pp. 566.

2. *The Practice of Medicine; a Treatise on Special Pathology and Therapeutics.* By R. DUNGLISON, M.D., Professor of the Institutes of Medicine in Jefferson Medical College, Philadelphia, &c.—*Philadelphia,* 1844. *Second Edition.* Two Volumes, 8vo, pp. 632, 684.

IN accordance with the intention announced in our former article on French and American Practical Medicine (vol. XVIII, p. 287,) we resume, nearly at the point where we left off, our parallel between the works of of MM. Dunglison and Valleix.

BRONCHITIS. M. Valleix recognizes three forms of this affection—the acute, the chronic, and the pseudo-membranous. Dr. Dunglison's division of bronchitis, properly speaking, is likewise tripartite—viz. into the ordinary acute bronchitis; epidemic acute B. (influenza); and chronic B.; whilst, as a singular variety of the chronic form, he introduces, under the name of "SUMMER BRONCHITIS," the affection commonly known in this country by the term of "hay-asthma," or "hay-fever."

M. Valleix subdivides one of his divisions (acute bronchitis,) into three forms, viz., the slight and apyretic, the intense febrile, and general capillary bronchitis.

Assuming that the signs of so familiar a disease as acute bronchitis are well known to our readers, we shall not consume their time by extracting from our authors the symptoms ascribed by such to the ordinary form. As might be supposed, their descriptions—excepting that that of M. Valleix is the more minute and detailed—correspond. We are, however, disposed to dwell on this writer's description of general capillary bronchitis. He considers that this very fatal form of the disease has been described under the names of *peripneumonia notha, exanthematica, catarrhalis, catarrhus suffocativus*, or *catarrhe suffocant* by many authors, such as Sydenham, Sauvages, Morgagni, Cullen, and Lieutaud. We must take the liberty of remarking that the *peripneumonia notha* of Sydenham appears, if not a different disease from the highly fatal one described by M. Valleix, to have affected a very different class of persons. Sydenham says expressly that peripneumonia notha affects those of adult age or beyond it, and who are addicted to the use of ardent spirits (Sydenham, ed. 1726, p. 209.) The description of Sauvages (Classes Morborum p. 500,) corresponds with that of Sydenham, in fact is derived from it, and equally represents a severe bronchitis affecting the aged, or those who have induced premature old age by intemperance. He considers it synonymous with "fluxion of the chest" (poitrine,) which identifies it with bronchitis. But the form of bronchitis which M. Valleix describes by the terms "general capillary" is emphatically a disease of childhood, so much so that his own experience for three years at the Salpêtrière and his researches into the practice of others have furnished him with only five unequivocal cases of this affection in adult and advanced life. We find in M. Valleix's description of the affection so graphic a picture of a disease to which, in spite of the most energetic and well-applied measures we have seen many a fair child succumb, that we present it with slight abbreviation to our readers:

"In general the disease commences as a common cold of variable intensity; and then we can discover at the posterior and inferior part of the chest on both sides, as in bronchitis of ordinary intensity, a subcrepitating rale extending to the third or the half of the height of the thorax. But soon these symptoms assume an unwonted intensity, and the sufferers present the following symptoms:

"The face is pale, the lips present a violet colour, the eyes are prominent, the countenance is extremely anxious, the nostrils are dilated at each inspiration; the patient is agitated and constantly changing his positions. Children somewhat advanced in life and adults sit and incline the head forward; or raise it by resting on their elbows. There is panting, violent dilatation of the thorax, and, at intervals, the breathing is sonorous and stertorous, but more tranquil after expectoration. The cough is violent and humid, recurring often in paroxysms, but does not possess any peculiar character.

"In very young children, there is no expectoration; in others difficult expectoration of a matter more or less thick, not frothy, and of a pale yellow colour, and of stringy mucus, occasionally frothy and streaked with blood. The voice is not affected; speech is brief and interrupted; there is violent pain under the sternum, and the oppression is extreme. The pulse is rapid, ranging in children observed by M. Fauvel, from 124 to 160.

"After a time varying in different cases, the strength beginning to fail, the cough becomes less vigorous, and the expectoration more difficult. The breathing is more embarrassed and constantly stertorous. The face and *conjunctiva* are highly injected. There is an expression of great terror, and the patient throws himself into singular positions, to avoid lying on his back, moving himself forward, lying on his stomach, hanging his head over the bed-side, or throwing himself forward to meet the air which he stands in need of. The pulse becomes 'wretched,' is increased in frequency, and often cannot be counted. There is constant somnolence, interrupted by momentary exacerbations, produced by a paroxysm of coughing, the act of drinking, or expectoration. Sometimes delirium and agitation are observable during the night, and death soon occurs."

After remarking that in this disease wheezes (râles) of various sorts are heard throughout the whole extent of the thorax, and that its sonorousness on percussion is rather increased than diminished, he goes on to say:

"If this description has been carefully studied, we shall easily discover that all the peculiarities which it presents are solely due to the great extent of the inflammation. We have, in fact, only symptoms of asphyxia caused by the difficulty of *hematosis* produced by the obstruction of almost all the air-passages, and by the impossibility that the air should come in contact with the mucous membrane through a layer of thick and glue-like matter." (Valleix, t. ii, pp. 76-8.)

We think this an accurate and expressive picture of one of the most fatal diseases with which infantile life is assailed.

M. Valleix gives a synoptical table of the diagnosis between his different forms of bronchitis and certain other diseases of the chest with which they might be confounded. We extract a portion of this:

| | |
|---|---|
| "I. *Febrile Bronchitis.* | I. *Pneumonia.* |
| Dull pain under the sternum. | Pain of the side in a situation more or less remote from the sternum. |
| Fever of moderate intensity. | Intense fever. |
| Subcrepitating wheeze at the inferior and posterior part of the chest on both sides. | Crepitating wheeze posteriorly on one side only. |
| Thorax normally sonorous. | Obscurity on the side where the abnormal sound is heard. |
| II. *Febrile Bronchitis.* | II. *Acute Phthisis.* |
| Pain under the sternum. | Pain under the clavicles or between the shoulders. |
| Normal sound under the clavicles. | Dullness in the subclavicular region. |
| Subcrepitating wheeze at the base of both lungs and posteriorly. | Crackling and subcrepitating wheeze at the summit of the lungs. |
| No hemoptysis. [We think this doubtful.—REV.] | Sometimes hemoptysis. |
| III. *General Capillary Bronchitis.* | III. *Exanthematous Fever.* |
| Frequently neither shivering nor vomiting at the commencement. | Shivering, vomiting, &c. at the commencement. |
| Neither coryza, nor watery eye, nor sore-throat, nor muscular pains. | Coryza and watery eye in measles, sore-throat in scarlatina, muscular pains in smallpox. |
| Subcrepitating wheeze diffused throughout the chest. | Subcrepitating wheeze null, or limited to a small extent of the base of the lungs. |
| IV. *General Capillary Bronchitis.* | IV. *Lobular Pneumonia.* |
| Dyspnea very intense. | Dyspnea less considerable. |
| Anxiety extreme. | Anxiety moderate. |
| Subcrepitating wheeze diffused throughout the whole extent of the chest. | Subcrepitating wheeze limited to a smaller extent of the lung." |

When we turn to the subject of the treatment of acute bronchitis, we find a complete correspondence, if not in detail, in the general proceeding recommended by our authors. M. Valleix's summary of treatment suggests for slight bronchitis, emollients, weak narcotics, mild purgatives, and revulsives; for febrile bronchitis, diluents, bloodletting (general, local, or both), narcotics, emetics, purgatives, spirituous and sudorific stimulants. These last are suggested on the authority of Laennec, but with much doubt and caution, and a suggestion from M. Mériadec Laennec, that the proceeding (with spirituous matters) is effectual only as a means of producing perspiration, and that this purpose is more surely and safely effected by an opiate taken warm. Instead of the stimulating potions of his uncle, Mériadac Laennec recommends that an ounce of syrup of poppies should be given in a cup of very warm pectoral decoction. For general capillary bronchitis, M. Valleix recommends, during the first period, bloodletting, emetics, revulsives, emollients, repose; during the second, light stimulants, and *strychnia* or *nux vomica.*

Dr. Dunglison's measures are, in substance, the same as those of M. Valleix. In mild cases of acute bronchitis, rest in bed, abstinence from animal food, and a diet of gruel, arrow-root, or tea, and mucilaginous medicines containing minute quantities of opium, are the means suggested. When the fever is considerable, the sense of heat and pain in the chest great, with much dyspnea, and violent and frequent cough, blood may be taken, and the operation may be repeated, when the patient is young and vigorous, more than once or twice. In very severe cases, the safety of the patient is said to repose on the vigorous employment of general bloodletting, followed by the application of cupping-glasses or leeches to the chest, and subsequently of blisters, and the contra-stimulant use of emetic tartar—on all the means, in short, which are demanded in acute laryngitis.

Dr. Dunglison approves of the declaration of Mackintosh, that in acute bronchitis, expectorants and diaphoretics are more injurious than beneficial, adding that the common error consists in the belief that there is any agent that can act either as an expectorant or as a diaphoretic, in all states of the system. Where it is necessary to encourage expectoration or diaphoresis, the pathological cause of the obstruction must be inquired into and removed. In this sentiment we fully concur, and would remark that the great power of emetic tartar as a diaphoretic and expectorant in acute bronchitis, depends on its correcting the local inflammatory condition and subduing the tone of the system constituting the resistance to the evacuations from the skin and air-passages.

INFLUENZA. Dr. Dunglison places influenza in this class, under the title of epidemic acute bronchitis. We think this as fitting a collocation as could be found for this singular disease, although the influence of its cause extends far beyond the bronchial mucous lining. He has collected from various quarters chronological lists of the different influenzas which have ravaged the world. There is considerable difference among his authorities, who are Most, Saillant, Webster, Zeviani, and Kluge. The list of Most is limited to the 18th century, and is therefore much less ample than the others. That of Zeviani is said to sin by excess, in consequence of the Italians attaching the name influenza to other epidemics besides catarrh. However this may be with respect to preceding centuries, we find this list omitting well-marked influenzas of the 18th century, for instance, the disease of 1775, described by Sir G. Baker and Dr. Fothergill. Webster's list comprises three centuries only, from the 16th to the 18th centuries inclusive; but for this period seems the most correct, excepting that the compiler appears to have multiplied epidemics by describing the same disease in different parts of its progress. He gives us, for instance, an influenza for each of those three successive years, 1788, 1789, 1790. By making some corrections, manifestly required, in Webster's list, and supplying from other sources the epidemics of the centuries preceding the 16th and those of the 19th century, the following enumeration of epidemics of influenza, which we consider if not absolutely correct to be very nearly so, is formed.

| | |
|---|---|
| 13th century . | 1239 |
| 14th „ | 1311, 1323, 1327, 1358, 1387, 1400. |
| 15th „ | 1410, 1414, 1438, 1482. |
| 16th „ | 1510, 1543, 1557, 1562, 1574, 1580, 1587, 1591, 1597. |
| 17th „ | 1602, 1610, 1622, 1647, 1650, 1655, 1658, 1675, 1679, 1688, 1693, 1697. |
| 18th „ | 1708, 1712, 1729, 1733, 1737, 1743, 1747, 1755, 1762, 1767, 1772, 1775, 1782, 1788, 1795, 1797. |
| 19th „ | 1801, 1803, 1831, 1833, 1837. |

We have endeavoured, in compiling this list, to avoid multiplying epidemics,—investing with plurality one and the same disease. But we cannot help feeling a doubt how far we have been successful in some cases. For instance, the last epidemic of the 18th century, or the first of the present one, marked by Kluge as having taken place in 1800, but which, according to our ideas, did not, at least in this country, become manifest till the year next succeeding, 1801, we find in Kluge's list followed by one in 1803. Now we cannot help feeling a doubt whether these two, inserted as distinct, were not the same disease continued in various parts of the world. A similar remark applies to the epidemics noted for 1831 and 1833. That of 1833 was manifest enough,—in fact, was little inferior in intensity and diffusion to the great influenza of 1837; whilst in certain districts of England there was no epidemic in 1831. Having repeatedly, however, observed that epidemics grow up slowly in communities, their seeds scattered here and there, requiring time for their perfect development and outbreak, we think it probable that an epidemic had been existing somewhere, in a slight degree, in 1831, which did not reach its height of diffusion and intensity till 1833.

Making every allowance, however, for confusion from this source, we have, during a period of five centuries and a half, epidemics of influenza at the rate of eight and a half per century. Now if we couple with this frequency of recurrence, the great amount of mortality induced by the disease, we find in influenza a formidable engine for abridging the duration of human life. It has been asserted that there is no notable increase of deaths during influenza, and Dr. Dunglison takes some pains to refute Dr. George Gregory's opinion expressed to this effect. We remember a clergyman, during the influenza of 1837, saying to us, "Some medical men tell me that influenza kills nobody,—*if this be the case,* I should like you to tell me, what is doubling the funerals in my parish?" Our reply was simply, "*this is not the case,*" for at that moment we were witnessing the ravages of the disease, especially among the aged and very young, to an extent quite appalling, around us. We find this proportion, the doubling of the mortality by influenza, existing abroad as well as at home. Thus in Hamburgh, in January 1836, there died 466 persons; in December of the same year, 364; and in January 1837, whilst the influenza was raging, 836 persons. This duplication of the mortality is during the height of the epidemic only, the duration of which is about one month. We could illustrate this from various quarters; but we select the returns from the Prospect Cemetery, Glasnevin, the largest in Ireland, for this purpose. The funerals here in January 1836, were 392; in December of the same year, 413; and in January 1837, 821. January, be it remarked, was, as far as we have been able to trace it, the period during which the influenza of 1837 was at its height in this country. When January, however, the month of the greatest intensity and diffusion of the disease was over, the funerals fell in number, there having taken place 537 in February, and 477 in March. If we add to this considerable immediate mortality the deaths which result from the direct sequelæ of the disease, such as hepatitis and abdominal dropsy, and from diseases of which the seeds may be in the system, but which owe their development to influenza, such as phthisis—and these diseases, with a fatal issue, we saw follow the influenza of 1837—we can understand that the fatality of an epidemic must be very great, of which the immediate mortality exceeded, in Europe, that from cholera.

Nearly eight years have elapsed since the last influenza, and another invasion of an epidemic, recurring eight times in a century, is in all probability not very remote. Has the experience of those which have passed furnished us with arms for combating that which may speedily follow? We question whether it has. M. Andral considers each epidemic of influenza as an individual unlike its fellows: "each epidemic has presented some special phenomenon; every form has constituted, as it were, a sort of morbid individuality, which, after its manner, ran through phases of increase and decrease, always commencing in the same way, and being invariably like unto itself." Dr. Dunglison, founding on the remarks of Andral, says:

> "If such be the diversity of phenomena in influenza, and such the uncertainty that hangs over it, it need scarcely be said that no fixed rules of treatment can be laid down."

These statements admit, we hope, of some qualification. Epidemics of influenza, although not necessarily identical, must be similar, or they could not be recognized as such. We have been close observers of two epidemics of this disease, those of 1833 and 1837; and, excepting in degree and diffusion, the latter being the more severe and general, they were the same affection, and we should say the same *principles* of treatment were applicable to both. To meet the very reasonable question,—what were these principles? we shall intrude a brief statement on our readers. We had before us patients suffering under inflammation of one or more sets of mucous membranes, and the diet, medical treatment, and general management were required to be such as this state of the system is well known to demand. In all cases repose in bed, and diluent diet, the patients being restricted to the simplest diluents with a little bread, were essential. In this restriction their own feelings led them cheerfully to acquiesce. In few instances of the primary disease was bloodletting performed; but when resorted to early, and this was done in some severe cases, it was beneficial. In relapsed attacks, in which the inflammation was generally of a much more decided and acute character than in the primary disease, this remedy was more required and better supported. Leeches to the thorax or abdomen, according to the seat of the more prominent local affection, were constantly resorted to, and with great benefit. The bowels were moved by means of gentle laxatives to which they were readily amenable, but they were found extremely intolerant of the stronger purgatives, which produced excessive pain, an effect which we ascribed to the inflamed condition of the lining of the digestive canal; apparently for the same reason it was observed that circumspection was required in the use of antimony. The large doses of Rasori or anything approaching to them, could not be borne, the vomiting, purging, and irritation of the bowels they occasioned being insufferable; but very small quantities of emetic tartar were introduced into the ordinary saline diaphoretics when such were given, or into the usual demulcents employed to allay cough. Of all remedies, however, well-regulated temperature and repose in bed were found the most efficient; at least without these, medicines were found of little avail, and all the relapsed attacks, which we witnessed in the two epidemics we have mentioned, were referrible to the neglect of these simple measures. Repeatedly have we seen rigors, followed by an exasperation of the thoracic affection, or an invasion of a fresh order of parts, such as the head or bowels, in all cases with more danger either immediately to life, or of laying the foundation of structural change,—result from the patient's quitting bed, and engaging in premature exertion.

In explanation of the extreme proclivity to relapse which exists in influenza, it should be remarked that there is a strong tendency in the disease to a natural cure by perspiration; after the disease has existed for a day or two, the skin is constantly wet. Now if this process be interrupted by premature rising from bed, not only are the efforts of nature marred, but, by the great evaporation from the wet surface, chill takes place, very speedily passing into rigor, with aggravation of the primary symptoms or excitement of a new train.

*Phthisis.* Certain important points in the diagnosis of chronic bronchitis and the ability with which they are handled by our author, lead us to touch on a disease, which on account of its familiarity we might otherwise have passed unnoticed. The diagnosis between phthisis and chronic bronchitis, is ably handled by both writers. We select M. Valleix's account of it as the most detailed and precise:

"Phthisis pulmonalis, when it has attained to an advanced period, presents no greater difficulties (than chronic pneumonia or pleurisy.) The dull sound under the clavicles, the gurgling (gargouillement,) or cavernous respiration, &c., leave no doubt of the existence of phthisis. But when this disease is only commencing, the physical signs are much less marked, and then there might be some doubts. We should then examine with care the general condition, and the local symptoms presented by the patients; and if there be emaciation out of proportion to the local symptoms, paleness, feebleness and night-sweats, we should suspect phthisis pulmonalis rather than bronchitis. If for a long time, the cough remains dry, or gives occasion to an expectoration of insignificant amount without any particular character, if there exist pain between the shoulders, considerable dyspnea, and a slight feverish movement towards evening, the certainty will be greater. Finally, if we ascertain the complete absence of the subcrepitating wheeze at the posterior part of the chest, and, on the contrary, under the clavicles some modification of the respiratory murmur, such as its feebleness on one side, a little roughness, prolongation of the expiration, or some crackling, we can no longer doubt the existence of phthisis and the idea of chronic bronchitis should be entirely rejected." (Valleix, t. ii, pp. 117-8.)

We pursue the subject of diagnosis, under the guidance of M. Valleix, into two diseases, which, when existing, are generally associated with chronic bronchitis, if not caused by it,—dilatation of the bronchi, and vesicular emphysema of the lungs. M. Valleix throws his diagnostic marks into the tabular form. We select for quotation those distinctive of dilatation of the bronchi from phthisis, confusion being most likely to occur between these diseases. In dilatation of the bronchi, notwithstanding that the disease be of long standing, emaciation is little observable; there are no nocturnal sweats; no hemoptysis; no habitual diarrhea; the physical signs may exist elsewhere than at the summit of the lungs; there is sometimes no indication from percussion, the sound of the chest being normal. In phthisis, there is emaciation dating from the commencement of the disease; there are nocturnal sweats; there is often hemoptysis; diarrhea is a frequent attendant; the seat of the stethoscopic signs is in the upper lobes, and there is a dullness on percussion or abnormal sound of the chest on a level with the tubercular cavities.

M. Valleix is brief on the subject of emphysema of the lungs, believing that the diagnosis is easy to any one who has properly studied this disease, a point on which we perfectly accord with him. Between this emphysema and phthisis, the following are his diagnostic marks. In emphysema, we find a rounded form of some parts of the thorax, or that it is generally dilated; the sound (on percussion) is clear on a level with the rounded portion; there is neither fever nor emaciation. In phthisis, there is neither rounding nor general dilatation of the thorax; the sound is obscure; there is hectic fever and emaciation.

The *treatment* of chronic bronchitis is discussed by our authors; by Dr. Dunglison succinctly, but without the omission of any material point, whilst M. Valleix spreads his details, with a prolixity too common to him, over the wearisome space of 23 pages. The former writer says our remedies must be regulated by circumstances; when there is much febrile and inflammatory excitement, the case must be treated as one of acute bronchitis, however long it may have existed. In ordinary chronic bronchitis, he considers the class of revellents the best. Intermittent counter-irritation by successive blisters, tartarized antimony, croton oil, or the liniment recommended by Dr. Graves, said to be an imitation of that of St. John Long, proves extremely serviceable. We annex the formula of this liniment, though not considering that any special benefit is likely to ensue from it beyond any other counter-irritant.

R. Olei terebinthinæ ℥iij.

Acidi acetici fortioris ℥ss.

Vitellum ov.;

Aquæ rosarum ℥iiss.

Olei limonis ʒj. M.

Emetics he deems very serviceable, not only in aiding mechanically the expulsion of secretion from the air-tubes, but preventing its too copious reproduction. The ordinary emetics of ipecacuanha and tartarized antimony are the most advisable. Of expectorants Dr. Dunglison does not speak very respectfully, objecting that he has been unable to observe any result from them, which might not be ascribed to their exciting effect on the stomach, and its extension to other parts of the system. We do not, we admit, see much force in this objection, which in fact does not apply to the curative effect of these medicines, but to their mode of operation. We have found benefit to arise from a proportion of the class of expectorants; but the advantage derived has not been of a nature to render the name, expectorants, suitable to them. They have done good by improving, as it has appeared to us, the condition of the bronchial lining, and diminishing the secretion of mucus from it, not by facilitating its extrusion; and this beneficial effect has appeared to arise from those medicines of the class which possessed a diuretic property, such as squill—an observation at least as old as Boerhaave.

The more stimulating remedies, such as tar-water, creosote, strychnia, balsam of copaiba, &c. are passed in review, but without Dr. Dunglison bestowing much praise upon them. Of tar-water, he says that he has administered it freely, and in cases in which the ordinary excitant expectorants are found to be serviceable, it has afforded relief; but further than this no advantage has accrued from its administration. The following is Dr. Dunglison's receipt for its preparation:

R. Picis liquid. ℥j; digere in aquæ O ij per dies octo et cola. Dose ℥ viij to ℥ xij in the day, mixed with milk.

In Germany, he says, the tops of the galeopsis grandiflora, a plant which is supposed to be the basis of a nostrum, celebrated in pectoral diseases under the name of "Blankenheimer thee," have enjoyed great repute. The following is the receipt for the preparation of the medicine from this plant:

R. Summitat. galeops. grandiflor.
Rad. althææ āā j℥.
Rad. glycyrrhiz. ʒij. M. A fourth part of this to be boiled in a pint and a half of water, and to be taken daily.

We think it probable that should Dr. Dunglison's work reach a second edition, he will give naphtha a place among the stimulant remedies of chronic bronchitis, and there only.

After some comments on the use of narcotics, and on that of stimulant inhalations, such as those of chlorine, which he says may be inhaled from a common dish by dropping any of the acids on a mixture of chloride of lime so that the acid may be disengaged slowly, he proceeds to consider the climates more suited to the subjects of chronic bronchitis. He thinks Santa Cruz in the West Indies, Hières on the shores of the Mediterranean, and Pensacola in the United States, suitable abodes for such invalids, possessing, as they do, a genial and agreeable climate.

The work of M. Valleix mentions, regarding the treatment of chronic bronchitis, only one remedy that is not noticed in that of Dr. Dunglison, and this remedy comes from the United States. It is the inspiration of cold air, recommended by Dr. Drake, of New York. According to this physician's method, the patient lying in a bath of 98°, or in a warm bed, inspires through a tube the atmospheric air when at a low temperature; or, when this is not the case, air cooled to 40°, Fahrenheit, by means of ice. Its author, Dr. Drake, says, that this remedy is beneficial, principally in warm weather; and M. Valleix hazards the reasonable supposition, that it is the warmth of the weather not the remedy that effects the amelioration.

MM. Blache and Chomel, who have commented on this practice, consider it to be suggested on merely theoretical grounds, and that experience has not as yet confirmed its efficacy: observing how much bronchitic subjects suffer from the inspiration of cold air, we should anticipate from it a different result from that which Dr. Drake professes to have observed.

Hay Fever. This singular and distressing affection is called by Dr. Dunglison "summer bronchitis," and is considered by him as a singular variety of inflammation of the mucous membrane of the respiratory apparatus. We learn from him that it exists equally in America as in Europe. Where a predisposition founded on idiosyncrasy exists, the effluvium of hay in flower may be an exciting cause of this disease; but other emanations, such as the odour of a rose, the bean-flower, &c. may produce it. The well-known irritating effect on the respiratory mucous lining of powdered ipecacuanha manifested itself in a young gentleman of Dr. Dunglison's acquaintance, by every symptom of hay-fever.

The best prophylactics, before the period of expected recurrence, are the cold shower-baths and the internal use of sulphate of quinine and sulphate of iron; but the disease once established, it will run its course, easily aggravated by low diet, much exposure, and debilitating measures generally; though it does not admit of much relief from any measures. A very sensible *lay* correspondent of Dr. Dunglison's, who had suffered from the disease, found some alleviation from prussic acid and black-drop, in small doses three times a day; quiet, "comfortable diet," and repose on a sofa rather than on a bed.

Pleurisy. Having shown the very great correspondence between our authors in their practice in several inflammatory diseases of the respiratory organs, we shall continue to trace their opinions through one other disease only of this class—pleurisy, and regarding this merely so far as the operation of paracentesis is concerned. With regard to this operation much doubt pervades the profession in this country at least, and this not merely in matters of detail, such as the precise place where, the time when, or the mode in which the operation should be done; but on the main fact, whether this operation is in any case expedient. We shall proceed to state the opinions of our authors on the question, which we regard as a very important one in all its parts, considering that it may be expedient to call in foreign counsel in a case when domestic opinion is much divided. By many, Dr. Dunglison remarks, all remedies in empyema have been deemed inoperative, and to this effect he quotes the authorities of Willis, Broussais, and Laennec; but at the same time he cites the more hopeful testimony of Dr. Stokes, who has recorded twenty cases of recovery by absorption.

When all remedies have failed and the patient is threatened with suffocation, or suffers *much distress from the mechanical pressure of the fluid on important organs*, the operation of paracentesis is the *last resource;* but he adds the important qualification, that absorption being often effected, even in unpromising cases, it has not always been necessary where it has been performed.

M. Valleix quotes largely from a thesis on the operation of empyema, published by M. Sédillot in 1841, containing an account of various operations performed, thirty-seven of which were in cases of pleurisy and non-traumatic pneumothorax. This writer says: "We are aware that

M. Gendrin attempted some years ago, at the Hôtel-Dieu, to apply the operation of empyema to acute pleuritic effusions; *but the constantly fatal result caused him soon to renounce this deadly method.*" Nevertheless, adds M. Sédillot, "should the effusion be considerable enough to bring on suffocation and menace the life of the individual mechanically by its own effect (à lui seul,) we believe that the evacuation of the liquid would give at least time to apply suitable treatment to the pleurisy, and would retard a fatal termination, which at the time was imminent." It seems scarcely necessary to argue on the hypothetical case put by M. Sédillot. What medical man has seen a case of acute pleurisy in which life was imminently threatened by the suffocation induced by mere mechanical pressure of effused fluid, and which said mechanical pressure and its effects could not be speedily relieved by bloodletting, and the other ordinary remedies of pleuritis? On this head, M. Valleix remarks, "That cases of this kind are excessively rare, *even if they exist,* and that there arises a difficulty, which we shall find recurring in another branch of this subject,—that of appreciating exactly the imminence of the danger. We consider, in fact, that threatening suffocation may appear of very different importance to different observers, and that consequently this sign will not serve to establish any positive rule." (t. ii, p. 549.) We have ourselves more than once been summoned to cases of acute pleurisy in the advanced stages, the earlier one having been neglected, that we might give an opinion on the propriety of resorting to the operation; in every case have we been adverse to it, and in favour of the continued employment of the remedies of pleurisy, bleeding, counter-irritation, mercury, diuretics, &c. and uniformly have our views been justified by the results.

Besides that of M. Valleix, we have the authority of Dr. Dunglison for the operation of empyema in acute pleurisy in the following terms: "the operation is not demanded solely in chronic pleurisy. If in the acute form, the effusion takes place so quickly and copiously as to endanger the very existence of the patient, owing to compression of the lungs and other important organs within the chest, it *may* be necessary." It will be observed that neither writer states of his own knowledge that the (apparently) hypothetical case put, has actually occurred, and has been relieved by paracentesis; indeed M. Valleix implies a doubt of the possibility of such a case. Another writer, M. Chomel,* between whose language on this point and that of Dr. Dunglison, such a correspondence exists, that it might be supposed that the one was derived from the other, is equally silent as to any experimental and practical grounds he has for his opinion. He expresses himself in the following terms:

> "Finally, in those cases (of acute pleurisy,) in which the different methods calculated to favour the absorption of the effused fluid have been employed in vain, and where the increasing difficulty of breathing obliges the physician to hazard a dangerous method rather than to allow the patient to perish of suffocation, we may and, in some cases, we ought to attempt the operation of empyema, in spite of the little success which generally attends it." (Dictionnaire de Médecine, article Pleurisie, p. 30.)

We have a great dislike to important practices being suggested on purely hypothetical grounds, and of the opinions we have quoted on performing

* Dictionnaire de Médecine; article Pleurisie.

the operation of paracentesis in acute pleurisy, that of M. Valleix comes the nearest to our own,—which is, that it should not be done. We now turn to the consideration of performing it in chronic empyema.

On this head M. Valleix says:

"We have seen in fact that simple pleurisy, which supervenes on a state of good health, tends naturally to a cure. If then we perform the operation of empyema in a similar case, how can we know whether our success is attributable to it or not? But in the cases contained in the work of M. Sédillot, it is almost always impossible to discover whether the pleurisy is simple or complicated. This is evidently a *lacuna* which ulterior observation can alone fill up, and which it is very important should no longer exist, because till facts have been looked at in this point of view, our discussions will be conducted without object and without profit.

"We have then, in reality, to guide us in our decision, only the persistence of the symptoms of pleurisy, emaciation more or less marked, occasional lipothymia, threatening of suffocation, &c., symptoms which we often find described in the cases of authors, and among others in those of M. Heyfelder. The occasion then for performing the operation remains to be decided by the judgment of the practitioner.

"Nevertheless there are certain circumstances which we may regard as counter-indications, and which it is important to indicate here. According to the summary of facts collected by M. Sédillot, there is not a single example of its success in aged persons. The oldest person in whom the operation was successful, was 40 years of age; all the other patients were young children, youths, or adults not above thirty. The duration of the *puothorax*, adds this author, is without contradiction, a circumstance unfavorable to the operation, which is owing to the atrophy of the compressed lung; yet this effect is not constant. Hippocrates fixed on the fifteenth day of the effusion, as the moment of the operation; but generally we can wait longer. Complications with *tubercles* and *pneumonia* are unfavorable; it is advisable, therefore, to ascertain the state of the organs, and especially that of the lungs. Nevertheless it is not shown that the existence of some tubercles is an absolute counter-indication to the operation; the main matter is, I repeat, to ascertain the circumstances in which this operation is *indispensable*, and this is the difficult point. Finally, all that we know is that in a considerable number (un assez bon nombre,) of severe cases, this operation has been crowned with success." (Valleix, t. ii, pp. 549-50.)

The questions seem reasonable, whether any proportion of the successful cases would have recovered without the operation; and, again, of fatal cases whether the fatality of some of them was not due to the operation. An affirmative answer to either of these questions would deduct materially from the recorded value of the operation. Our own opinion is, that in cases of simple pleurisy occurring in persons in previous good health,—cases which according to M. Louis terminate almost uniformly in recovery, the operation will not only not be required, but if performed be injurious. In another class, constituting a great majority of the cases of chronic pleurisy, those in which the general cachectic aspect of the patient leads to belief of the existence of tuberculation or other disease on which the effusion is dependent, whilst we admit that we have been more successful in their treatment without the operation than with it, and that, consequently, we have, for some time, resorted to it solely as a means of relieving dyspnea and distress, not as a method of cure, we would yet acknowledge that the mass of evidence adduced in its favour by Dr. Hamilton Roe* is such as

* Medico-Chirurgical Transactions, vol. ix, p. 199, et seq.

will lead us at least to reconsider our opinion, and it may be to modify our proceedings. Whilst we say this much, and it is no more than it deserves, for Dr. Roe's paper, we yet take the liberty of questioning whether certain of the cases he quotes of inflammatory hydrothorax, by which term is meant, we presume, pleurisy with non-puriform effusion, would not have done at least as well without the operation as with it; in this questionable category the nineteenth case, among others, appears to stand.

DISEASES OF THE HEART. Diseases of the circulating system receive, as they merit, ample attention from our authors, occupying about 90 pages of the work of Dr. Dunglison, and one entire volume of above 600 pages in that of M. Valleix. Their account of particular diseases is prefaced by both writers with some physiological remarks on the heart's movements and the sounds with which they are associated.

There is some difference of opinion between British and French physiologists on these points, and M. Valleix presents us with a long disquisition on this controversy, if such it can be termed. After an exposition of the views of the London and Dublin Committees, and a very long statement indeed of the sentiments of various French writers on the same subjects, he gives his verdict in favour of the exposition of the movements of the heart, and the explanation of the sounds with which they are associated, published by M. Beau in the 'Archives Générales de Médecine.' The following summary of the physiological condition of the heart comprises, with other matter, M. Beau's account of the order of its movements, and the sounds with which they are associated.

"1. *Situation of the heart.* A line traced parallel to the upper edge of the third rib corresponds nearly to the base of the heart and the arterial orifices. Its point beats from two to three inches below the nipple. The right ventricle is situated in part under the sternum, but still more to the left of this bone; the left ventricle is entirely to the left of it. The ventricles, covered in great part by the lungs, are exposed only to the extent of from two to three inches.

"2. *Volume of the heart.* This augments in proportion as we advance in life. It is smaller in the female sex.

"3. *Orifices of the heart.* These are dilated in proportion as we advance in life. They are larger in men.

"4. *Movements of the heart.* 1°, Contraction of the auricles, dilatation of the ventricles, contraction of the ventricles succeeding each other without interval; 2°, rapid influx of the blood into the auricle, then slow continuation of this influx, and at the same time repose of the ventricles; these new series of movements in the same order.

"5. *Sounds of the heart: First sound,* due to the shock of the blood thrown by the auricle into the ventricle, prolonged during the dilatation, and consequently after the passage of the blood through the auriculo-ventricular orifice; second sound, due to the shock of the blood, flowing suddenly from the veins, against the parietes of the auricles, and corresponding to this influx.

"Accessory sounds (bruits de renforcement); ventricular contraction; shock produced by the reflux against the sigmoid valves, of the blood thrown into the large arteries; striking of the heart against the thorax." (Valleix, tome iii, pp. 33-4.)

Dr. Dunglison's views accord with those of English physiologists in general, viz., that the first sound is referrible to the contraction of the ventricles, and the second to the obsticle presented by the semilunar valves to the return of the blood from the arteries into the heart. He considers these views to have been shown to be, in all probability, correct, by the recent researches of competent observers, Messrs. Pinnock and Moore of Philadelphia.

The opinions of the British physiologists appear to us to exceed those of M. Beau in clearness and simplicity. The difference between these authorities consists in this, that what are considered by the former writers as the causes of the sounds, ventricular contraction in the case of the first sound, and the closure of the sigmoid valves against the refluent blood in the artery in that of the second, are regarded by M. Beau as merely producing accessory sounds; whilst his principal causes are in both cases of the same nature, shock of blood against the ventricular parietes from the auricle in the first sound, the same shock against the parietes of the auricle from the veins in the second. Without professing to decide, where so many doctors disagree, we would merely venture to remark, that the first sound is agreed by almost all to be synchronous with the systole of the ventricle, which is inconsistent with the cause assigned for it by M. Beau; and that in the case of the second sound, the sharp, clacking nature of this sound would naturally suggest the quick closing of a valve, and not the flow of blood from one cavity into another.

We proceed to compare our author's views of certain diseases of the circulating system, and first of pericarditis.

*Pericarditis.* Dr. Dunglison regards acute pericarditis, when occurring uncomplicated, and with all its characteristic phenomena, to be as easily recognizable as any internal disease. These phenomena are as follows:

"After the occurrence of the ordinary evidences of internal inflammation, an acute, pungent or lacerating pain in the region of the heart, under the sternum, which extends towards the epigastrium, and sometimes between the shoulders; a sense of more or less oppression, giving rise to rapidity of breathing, often amounting, in the progress of the disease, to panting; palpitation, irregular, jerking or intermittent pulse; dull sound over the region of the heart; at times, a sound as if two dry surfaces were rubbed against each other, or of the cracking of new leather (*bruit de frottement*, and *bruit de cuir neuf*,) without the existence of any sign of inflammation of the lung or pleura. Incapacity of lying on the left side has been pointed out as amongst the functional derangements most commonly present, (Joy;) but the author has observed it to be very frequently wanting." (Dunglison, vol. i, p. 467.)

We must not, however, always expect symptoms thus clearly marked, or an uncomplicated disease, and we resort to the work of M. Valleix for a more detailed symptomatology of pericarditis to aid us should some of the signs mentioned by Dr. Dunglison be found wanting, pain for instance, which is, according to MM. Louis and Bouillaud, null or very slight in half the cases; or, should complication increase the difficulty of our diagnosis.

M. Valleix relies much as a diagnostic mark on a projection or *vaulting* of the precordial region, pointed out by M. Louis, and which is considerable in proportion to the extent of the effusion. This projection is of an oval form, its longest diameter being from above downwards. It is bounded above by the third or second rib, and below by the eighth or ninth. When the effusion is rather considerable, the intercostal spaces are widened by the raising of the ribs, and their concavity is effaced, or even is replaced by a slight convexity.

Percussion performed over the precordial region returns a dull sound to an extent rather beyond that of the manifest projection or vaulting. This dullness is at its *maximum* about the middle of the projection, that is to say, on a level with the fourth rib, where there is felt a very marked resistance, observed principally by M. Piorry. This author has likewise remarked that the dullness in sound is heard over a less broad surface in proportion as we proceed in our examination from below upwards; and that it in some degree changes its place, (as might naturally be conceived from its cause,) according to the position of the patient. By auscultation we perceive that the respiratory murmur is completely *null* in the centre of the precordial region, but that it becomes audible, though distant, in proportion as we approach the circumference of the effusion. The sounds of the heart are heard at a distance, unless the effusion be very inconsiderable. They are more distinct on a level with the third rib, or little below it, than in the centre of the precordial region, which proves that in pericarditis the heart is somewhat lifted above its ordinary position. They are generally not affected in character; but they are more dull, more difficult to perceive than in the normal state, which arises from their remoteness from the ear, and the interposition of fluid. In some cases there is heard a bellows-sound, totally distinct from any of the pericardial noises to be afterwards adverted to. M. Bouillaud ascribes this either to endocarditis producing thickening of the valves, or *coagula* in the interior of the heart. Dr. Hope ascribed it to augmentation of the intensity of the pulsations of the heart.

The pericardial sound first pointed out by M. Collin under the name of "sound of new leather," (des Diverses Méth. d'expl. de la Poitrine, Paris, 1824,) was rarely observed for some years after its discovery; but this seems to have arisen from parties expecting a noise precisely similar to that adopted by the discoverer to illustrate the character of the cardiac sound, whereas there is much variety in the sound, and it may be, in many cases, more aptly compared to the noise produced by rubbing two surfaces together, to that of rasping, or of parchment, &c., than to that in which M. Collin first found its similitude. It is always very superficial, and ordinarily accompanies both sounds of the heart; but is generally stronger during the first than the second sound. M. Collin thought it was owing to the dryness of the serous membrane before the effusion took place; and it is the fact that when the accumulation of serum becomes considerable this symptom disappears, though it may have previously existed to a very great degree. The general opinion, however, now is, that it depends on the friction against each other of two false membranes, more or less rough and dense.

On the subject of causes of pericarditis, M. Valleix remarks that the coincidence of inflammation of the pericardium with acute articular rheumatism is a remarkable fact, which, although indicated by Sydenham (where?) and later by M. Chomel, has been better established by M. Bouillaud.

This is one of the small traits of nationality of which other examples are to be found in the work of M. Valleix. Why, the connexion between rheumatism, pericarditis, and other affections of the heart has been familiarly known to British practitioners from the early part of the present century, and was mentioned *by at least three writers of this country prior to any publication of M. Chomel on the subject:* viz., Dr. Davis of Bath, in 1808; Sir David Dundas (Med. Chir. Trans. vol. i,) in 1809; and Dr. Wells (Trans. of a Society for promoting Med., &c.) in 1812; whilst M. Chomel's work on Rheumatism was published in 1813, and the article on the heart in the 'Dictionnaire de Médecine,' the conjoint work of himself and Béclard, in 1822. In a work published by M. Bertin on Diseases of the Heart, in 1824, of which M. Bouillaud, to whom so large a share in establishing the connexion in question is ascribed, was the editor (redacteur), not a word is said of any connexion or coincidence between rheumatism and affections of the pericardium or heart!

The treatment advised by Dr. Dunglison comprises, as might be presumed, bloodletting freely practised so as to make a decided impression on the system, and to be repeated if the state of the patient render it necessary, along with cupping or the application of leeches. Simultaneously with the bleeding, he gives a solution of tartarized antimony combined with tincture of digitalis (half a grain of the former medicine and ten drops of the latter,) four times a-day. He was of opinion that a very serious case of rheumatic pericarditis, combined with endocarditis, was cured by the "*sedation*" induced by the acetate of morphia, causing insupportable nausea, which did not entirely cease for forty-eight hours. Should the disease not yield speedily, he would employ mercurials, giving two grains of calomel, and one eighth of a grain of acetate of morphia, every four hours.

The proceedings advised by M. Valleix do not differ materially from those of Dr. Dunglison as to the mere depletory parts; when he comes, however, to consider the administration of mercury he finds himself unable to decide between the opinion of Dr. Hope, whose mode of administering the mineral in this disease he quotes at considerable length, in its favour, and those of M. Gendrin, who has no confidence in this mode of medication. "How to decide," he asks, "in this clash of opinions? If we had from both parties facts in support of opinions we might arrive at some conclusion; but between two simple assertions it is impossible to decide; and this is a fresh proof of the bad method employed in the study of therapeutic questions."

Thus far we would agree with M. Valleix, that a return from each party of cases treated with mercury, comprising the cures and the failures, would have been of much value towards the decision of the question, although admitting that our own opinion founded on observation of the effect of mercury on pericarditis and other inflammation of fibrous membranes would lead us to coincide, in this respect, rather with Dr. Hope than M. Gendrin.

Most writers, such as Hope, Bouillaud, &c., recommend blisters to the precordial region to promote absorption when the febrile symptoms begin to subside. But M. Gendrin relies upon them as a main curative means, suggests that they should be multiplied according to the violence of the disease, and that they should always be of great size. The same writer advocates (perhaps not with much consistency,) the employment of sedative topical applications, especially very cold water or ice when the pains are very extreme, the palpitation very violent, and the anxiety great. M. Gendrin, however, regarding the safety and innocence of this mode of medication as not absolutely demonstrated, recommends great prudence in its employment, whilst M. Valleix says that we ought to act with so much the more circumspection as these means are of very questionable value. The remarks of the author of the practice taken in conjunction with those of his commentator would entirely lead us to let it alone altogether.

ENDOCARDITIS. We turn now to the subject of endocarditis, which is treated of by both our authors, but under different names, for Dr. Dunglison, considering that the endocardium cannot be affected with inflammation without the muscular substance of the heart participating, treats of it under the head of carditis, whilst M. Valleix considers it, and we think properly, as an independent disease, discussing the very rare disease, carditis, apart. We question whether Dr. Dunglison might not, on the grounds he has assigned in the case of endocarditis, have considered pericarditis also under the head of carditis.

M. Valleix considers that the symptoms of endocarditis are very obscure and inconstant; that of those assigned to this disease a proportion may be absent in any given case, and that those present may arise from another disease. The following are, according to him, the positive signs of acute endocarditis: it ordinarily arises in the course of an acute disease; it is attended with uneasiness, pain in the precordial region (this symptom is often wanting,) and anxiety; dullness of sound to a small extent on percussion; considerable (notable) augmentation of impulse; sounds of heart obscure, the pulsations being somtimes irregular; bellows or rasp-sound. According to Dr. Dunglison, the bellows-sound is one of the most characteristic and constant of the phenomena of endocarditis, and is at times so great as to mask one or both of the sounds of the organ. The impulse of the heart against the chest is generally so forcible and superficial as to be distinctly seen and readily recognizable by the hand. The pain is commonly very slight, and in many cases he has observed none whatever, nor is the dyspnea urgent. The *dullness of sound on percussion* in endocarditis is distinguished by both writers from that attending pericarditis by its greater extent in the latter case, whilst the *pulsations of the heart* are very forcible and superficial in the former disease, indistinct and remote in the latter.

Both writers divide the disease into the acute and chronic form, and they may be said to be in accordance as to its treatment, for Dr. Dunglison says it ought to be the same as that of pericarditis, whilst M. Valleix, without employing this expression, mentions the same remedies and passes similar comments upon them, as when discussing this disease.

HYPERTROPHY OF THE HEART. That too familiar disease, hypertrophy of the heart, receives much attention from our authors. The subject of its etiology, which we cannot help thinking a very obscure one, is considered by each but in a different spirit, for Dr. Dunglison seems much more satisfied with the mechanical causes ordinarily assigned than does M. Valleix. The mechanical causes mentioned by the former writer are affections of the orifices of the heart narrowing them and giving occasion to more powerful contraction of its muscular fibres, insufficiency of the valves permitting reflux of blood, and narrowing of the arteries emanating from the ventricles, by fibrinous or ossific matter. This last condition the author considers to be much more frequent after acute rheumatism. Among causes of a different description he notices inflammation of the pericardium, palpitation and too much action of the heart however induced.

M. Valleix looks at these matters with a more scrutinizing eye. M. Beau having found many cases of hypertrophy in which adhesion of the pericardium, so strait that the play of the valves of the heart might be impeded by it, existed, has considered such adhesion a cause of this disease, and similar observations have been made by Dr. Hope. Certain unedited researches, however, submitted by M. Louis to our author, prove that adhesion of the pericardium to the heart has not been met with in any considerable degree more frequently in diseased than in healthy hearts. In reference, too, to a supposed cause, mentioned by Dr. Dunglison, as it had often been by others, contraction of the cardiac orifices, M. Valleix again quotes Louis to show that the original observations of this excellent writer, and those which he has taken pains to gather from others, demonstrate that in three fifths of recorded cases there existed no connexion between lesions of the lining membrane of the heart, either at the orifices or other points of its entire extent, and the production of hypertrophy either with or without dilatation. Against a very common supposition that an obstacle opposed to the circulation by any chronic disease of the lungs is an ordinary cause of this affection, we have the high authority of Louis, conjointly with that of M. Bizot. These gentlemen have found in the phthisical, whose lungs were so studded with tubercles as to be scarcely permeable to air, the heart, so far from being hypertrophied, considerably diminished in volume. The same result ensues when a cancerous formation is opposed to the passage of the blood through the lungs. M. Valleix considers pulmonary emphysema to be at once a predisposing and exciting cause of hypertrophy of the heart, as the researches of M. Louis have shown. (Rech. sur l'Emph. M. de la Société d'Obs. t. i.) Should emphysema of the lungs exist for a certain number of years, this disease is sure to display itself, without any other especial cause, and to make more or less rapid progress. After some remarks on what are considered mental causes, and such circumstances as violent exercise, and addiction to spirituous liquors, and after censuring the phrase of Dupuytren, "*nutritive irritation,*" and we think justly, for it is a mere pompous expression, not an explanation of the fact, that hypertrophy often arises without *assignable cause,* he concludes with the true remark that the subject requires more investigation than it has received.

We do not feel disposed to follow our authors through a field so much trodden as the symptoms of this disease or its influence on other organs besides the heart, or through certain discussions on the propriety of the division of this affection, generally adopted since the publication of the work of M. Bertin, into simple, concentric, and eccentric, but proceed to consider their remarks on treatment.

Bloodletting is the first remedy mentioned by both; but neither of them would carry it to the extreme recommended by Valsalva and Albertini for aneurism, under which these writers classed the affection in question, especially when it was associated with dilatation, or (to use another expression) when of the eccentric form. Dr. Dunglison would limit to one his general bleeding, preferring after this to have the conjoint benefit of the depletion and revulsion of leeches to the precordial region. But M. Valleix, although not neglecting local bleeding, either from the precordial region, or, should ingestion of the liver demand it, from the vicinity of this organ or the anus, allows repeated general bleedings. He, moreover, dissents from a caution given by Dr. Hope against any considerable bloodletting where much dilatation is associated with the hypertrophy, alleging abundant and recent authority for its beneficial effects under such circumstances.

Both our writers recommend abstemious diet (though neither of them would push abstinence to the extreme of Valsalva or Albertini) and repose; indeed a better summary of diet and regimen for this disease cannot be anywhere found than is presented to us by M. Valleix. These are his words:

"Take only a small quantity of light food; abstain from wine and strong liquors; avoid violent mental emotions, and take only moderate exercise and especially passive exercise."

Sedative medicines find a place, of course, among the means recommended by our authors, and digitalis is one of these remedies suggested. Dr. Dunglison associates it with hydrocyanic acid, M. Valleix (on the authority of Goelis) with laudanum and succinate of ammonia, especially in the palpitations of children. The former places among remedies for this disease of the sedative class, acetate of lead given in conjunction with the sulphate or acetate of morphia, (two grains of the acetate of lead with one third of a grain of either of the other salts three times a day,) but he admits that this combination is less efficacious than the other sedative medicines he has named.

Diuretic and cathartic medicines find a place among the remedies of hypertrophy mentioned by our authors; but they both, without expressly saying that their employment should be limited to cases where hydropic symptoms are associated with the main disease, declare that there they find their most appropriate application. Among the diuretics, equally as among the sedatives, digitalis holds the first place with both writers. Dr. Dunglison advises that it should be combined with calomel, squill, or colchicum. Among cathartics, M. Valleix recommends camboge, aloës, jalap, and extract of colocynth; whilst Dr. Dunglison mentions with commendation (on the authority of Dr. A. T. Thompson), that first of hydragogues, elaterium, which finds no place in the *codex medicamentorum* of M. Valleix.

This last writer concludes the section of his work now under consideration, by passing in review what he calls the medicines intended to act directly on the hypertrophied tissue. This portion of his work instantly attracted our earnest attention, for we had long been anxious to discover something capable of fulfilling so laudable an intention. When we found, however, an enumeration of sundry mineral waters, such as those of Vichy, Carlsbad, Ems, and Seltzer, and, besides, were assured, on the authority of Kreysig, that copious potations of pure water must be very efficacious in hypertrophy because they cured diseases so analogous to it as schirrus and cancer (!), the feeling of hopefulness with which we commenced the perusal of the paragraph was by no means augmented.

*Dilatation of the heart.* This also obtains notice from our authors; from M. Valleix as an independent disease, if he can be considered as at all admitting its existence; but from Dr. Dunglison, as a condition of atrophy of the organ. M. Valleix says it is so rare that M. Louis has not met with a single case of it, and, though William Hunter, Lancisi, Burns, Corvisart, and Laennec have mentioned examples of it, unfortunately, whilst they have said that the heart was dilated, and its parietes attenuated, they have not entered into details sufficiently ample to permit others to form a precise opinion.

We hold that this is not only an existing disease, but one of much more frequent occurrence (at least in the country) than the sceptical tone of M. Valleix would lead any one to suppose. Its symptoms may be stated to be: Dullness on pericardial percussion over a considerable space; feeble impulse of the heart; its sounds *normal,* very clear, and distinct; feeble, soft, and compressible pulse; great tendency to coldness of the extremities; stasis of blood in the venous system, and an extreme tendency to anasarca.

The treatment of this form of cardiac affection recommended by M. Valleix is by tonics, especially preparations of steel; but he adds, we must not hesitate to bleed, should the stasis of blood be considerable. Our experience tells us that there is no disease of the heart in which the difficulty of affording relief is greater than this; we have ever found hypertrophy and its complications more susceptible of amelioration. A course of tonics, and, at the same time, giving freedom to secretion by hydragogue purgatives and diuretics, we have found the most safe and beneficial proceedings. Bloodletting, though it may afford some temporary relief, is generally a measure of questionable propriety.

The views of our authors on the treatment required where the cardiac valves are affected admitting of inference from the sentiments they have promulgated on other diseases of the heart, we shall not pursue their labours into this branch of the subject, neither do we purpose passing in review their opinions on diseases of the blood-vessels. Having, therefore, brought to a close the notice we intended bestowing on the portion of their works which has reached us, it remains that we should express an opinion of their respective value. They are both of them good books, but of different kinds, for whilst the work of Dr. Dunglison is a hand-book of easy practical reference, apparently intended for the guidance of the youthful and inexperienced; that of M. Valleix consists of a series of complete treatises on diseases, comprehending what may be termed our present knowledge of the subject, and showing the various modes of practice inculcated by, at least, the best French authorities of the day. Both will prove valuable additions to any library, for the knowledge seized for immediate use from the pages of Dr. Dunglison may be subsequently "*supplemented*" from the more elaborate descriptions of M. Valleix.

